# Supplementary material for: Two novel genomes of fireflies with different degrees of sexual dimorphism reveal insights into sex-biased gene expression and dosage compensation
Source: Commun Biol. 2024 Jul 27;7:906. doi: 10.1038/s42003-024-06550-6 (PMC11283472; doi:10.1038/s42003-024-06550-6)
Supplement: Supplementary file 2 — Supplementary Information [file 42003_2024_6550_MOESM2_ESM.pdf]

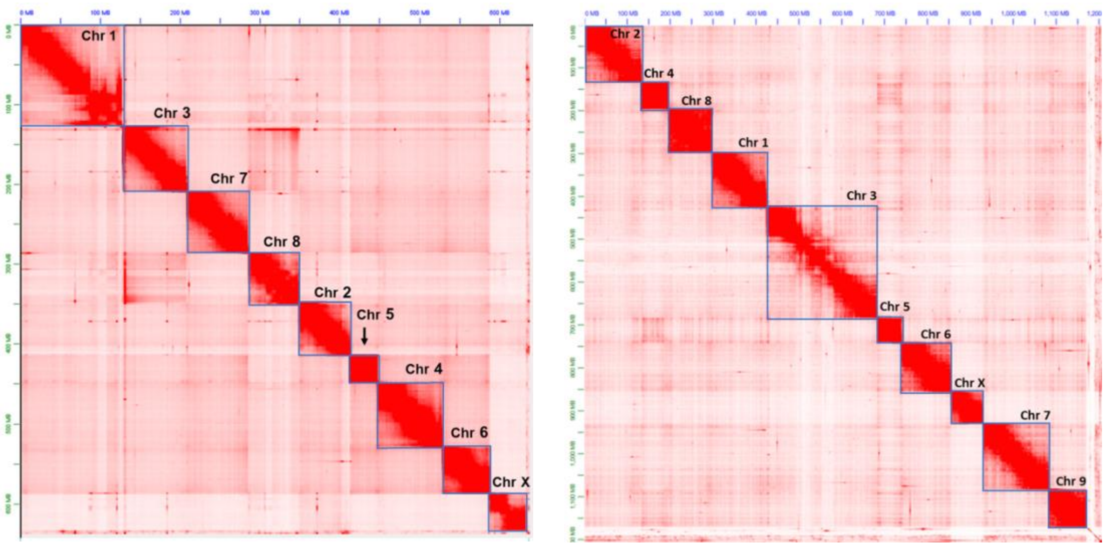

Figure S1. Juicebox HIi-C contact heatmaps. Left panel: *L. splendidula*. Right panel: *L. italica*.

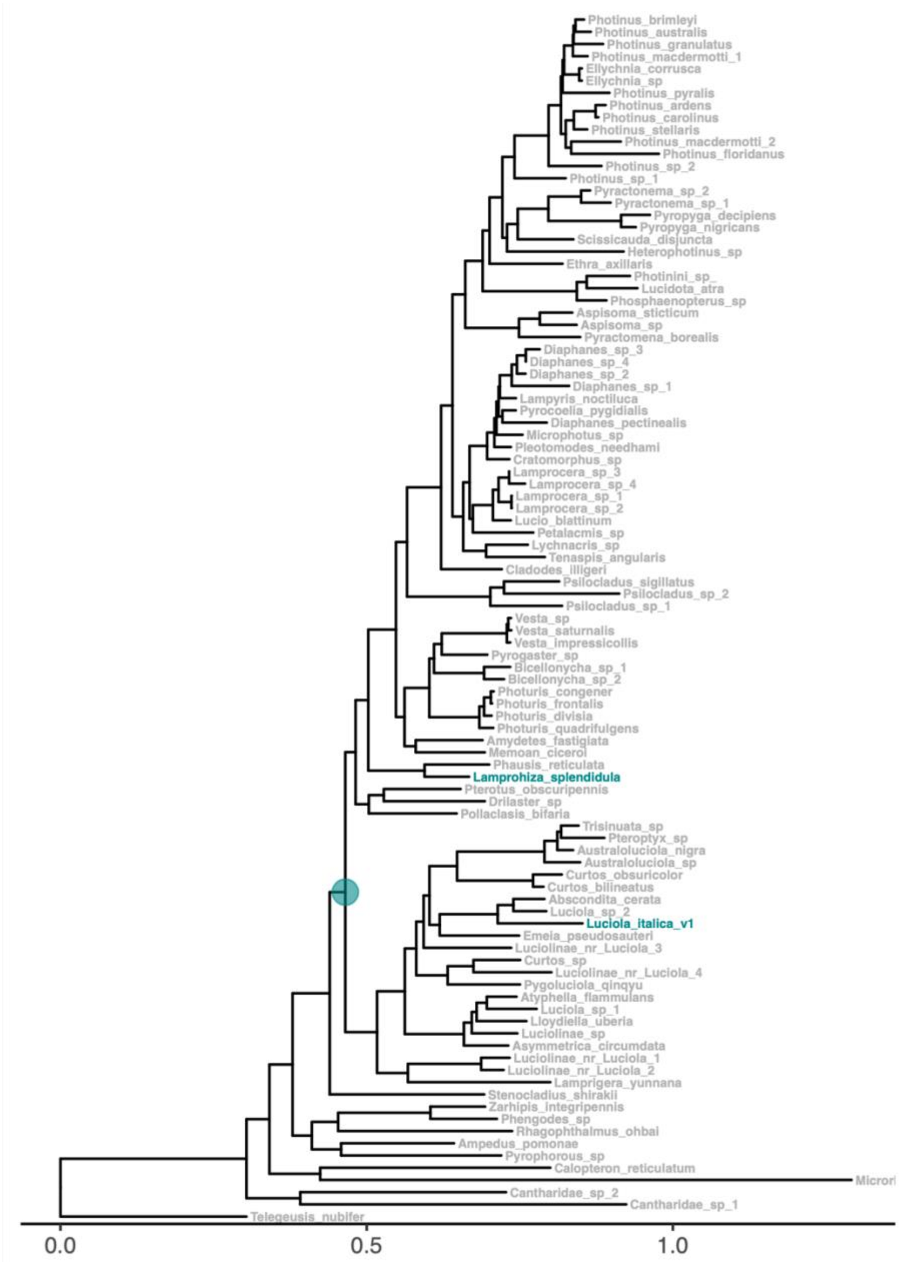

Figure S2. Phylogenetic placement of *Luciola italica* using a subset of the AHE dataset generated in Martin et al. (2019). The x-axis shows nucleotide divergence and the blue circle indicates the node of divergence between *L. italica* and *L. splendidula*, which is estimated to be around 140 million years ago (Höhna et al. 2021).

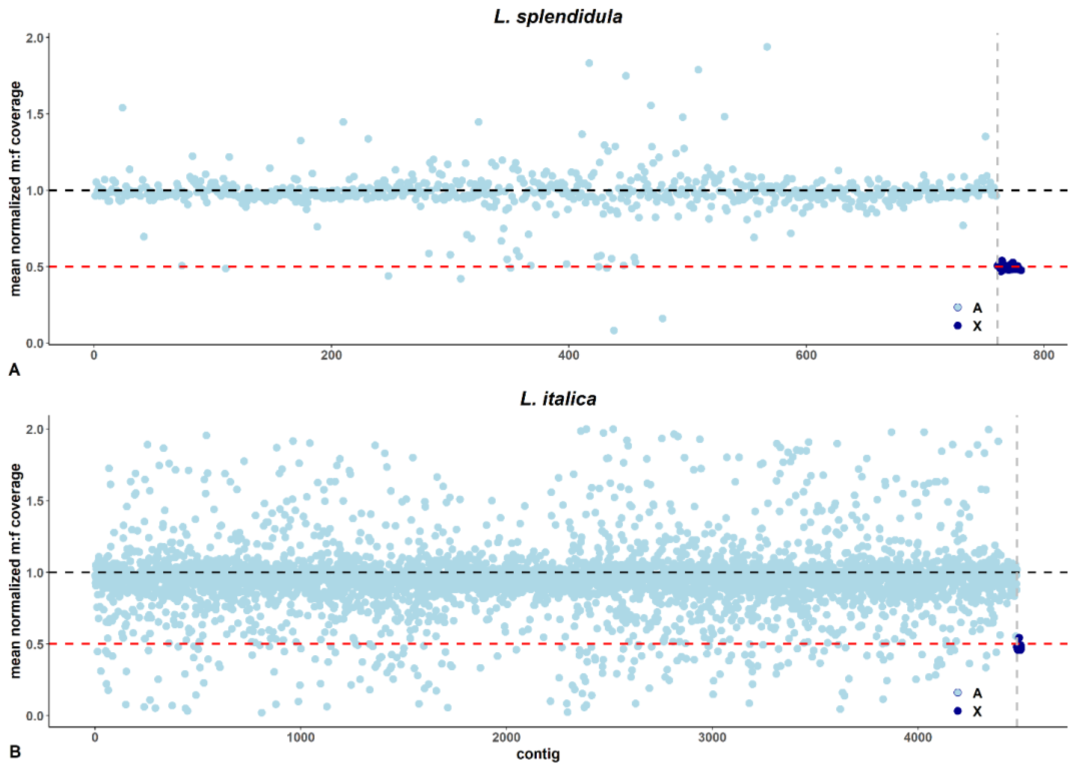

Figure S3. Identification of the X chromosome. Mean normalized male to female coverage ratio per genomic contig. A) *L. splendidula* B) *L. italica.* Shown are only contigs larger than 30kb and used a Wilcoxon Sum Test to identify coverage not deviating from m:f = 0.5. X-linked contigs are colored in dark blue and autosomal-linked contigs are colored in light blue.

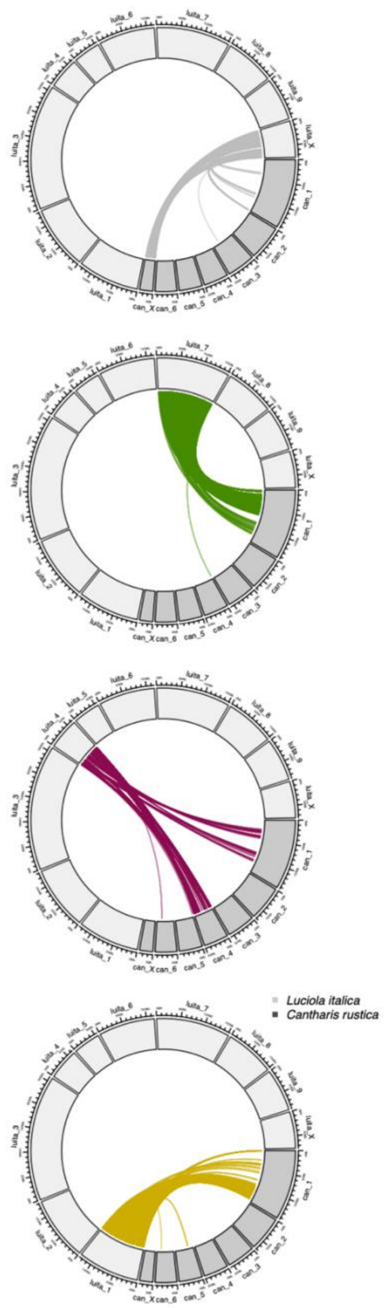

Figure S4. Genome synteny per chromosome of *L. italica* against *Cantharis rustica*.

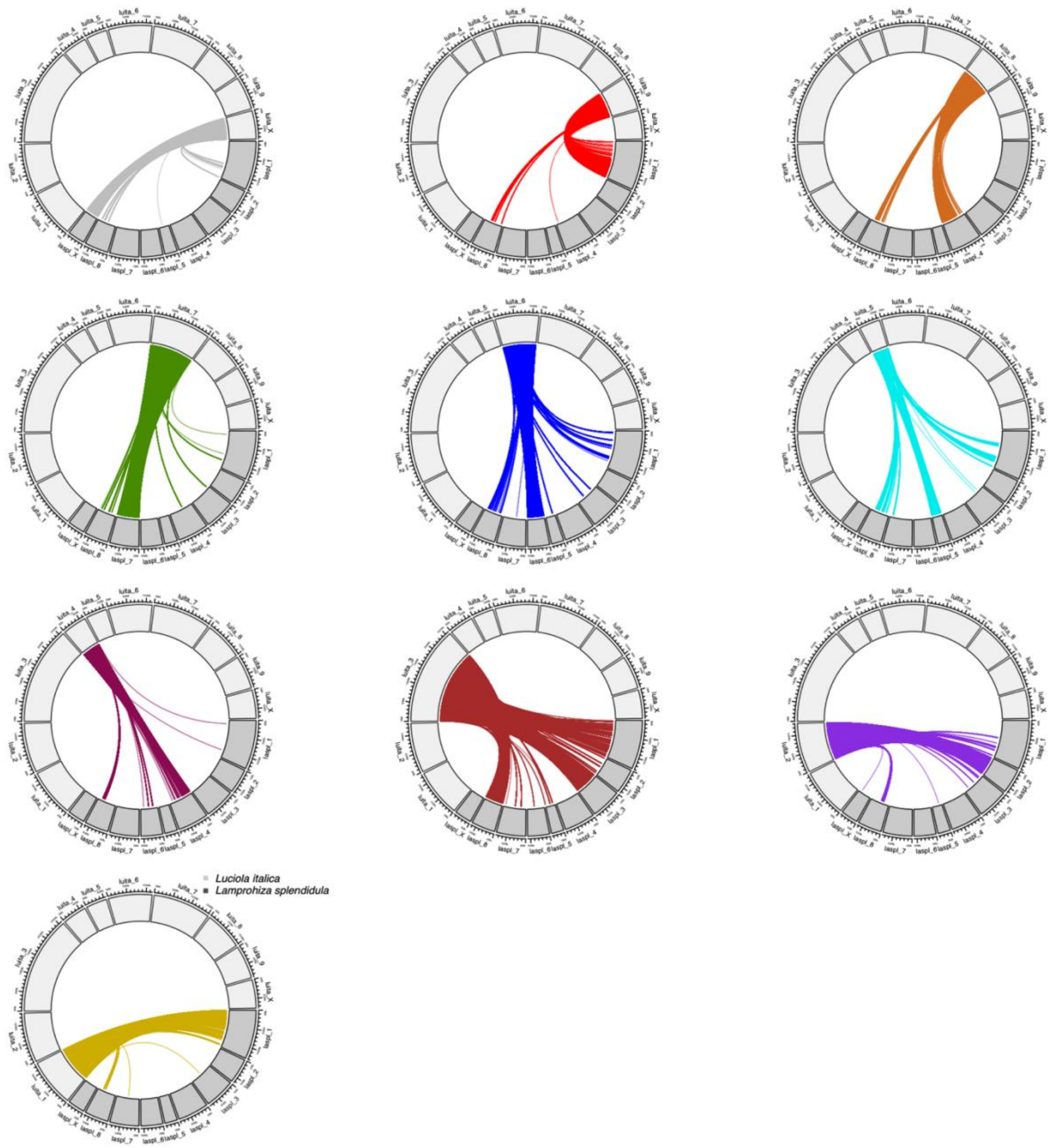

Figure S5. Genome synteny per chromosome of *L. italica* against *L. splendidula*.

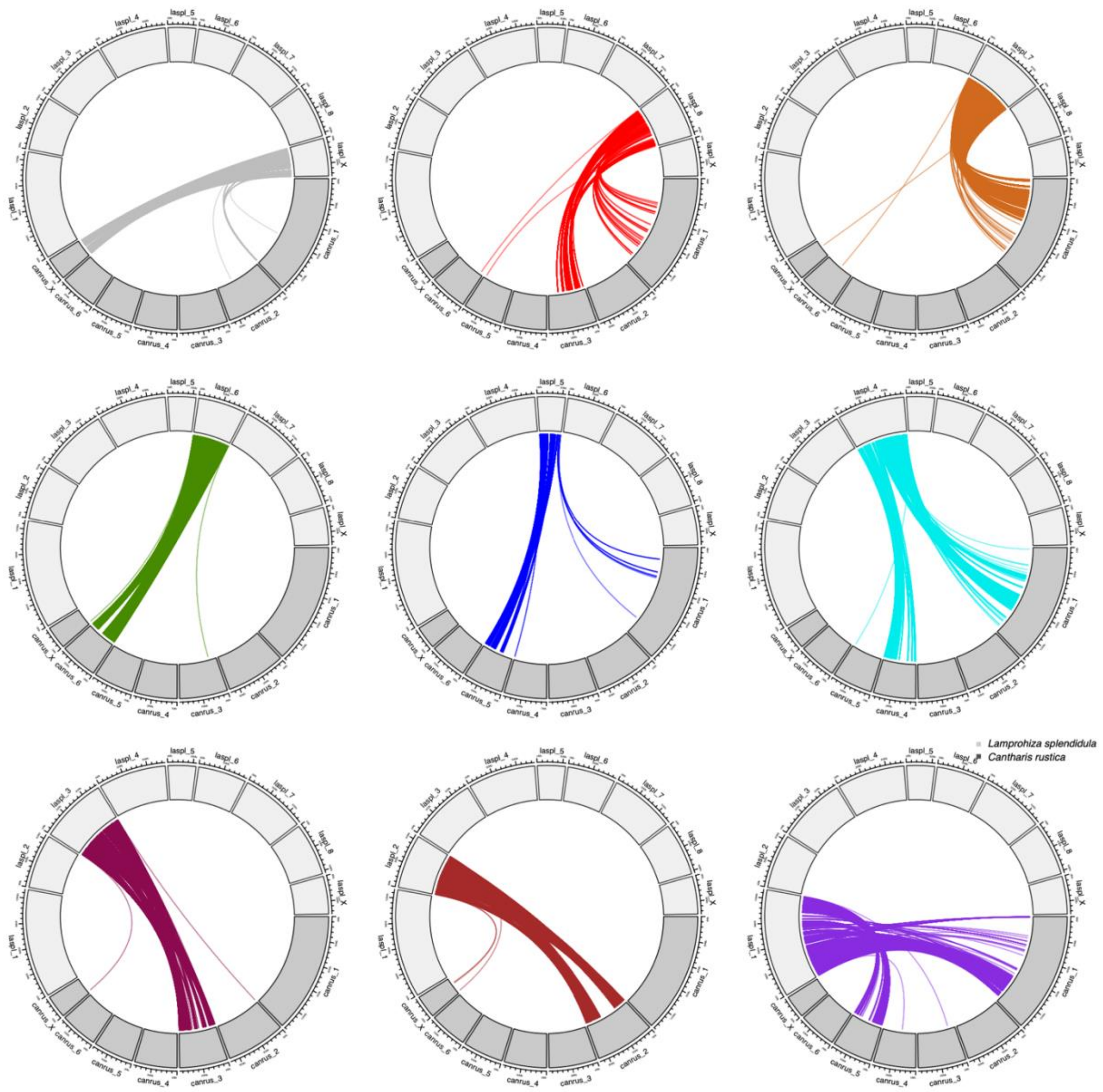

Figure S6. Genome synteny per chromosome of *L. splendidula* against *C. rustica*.

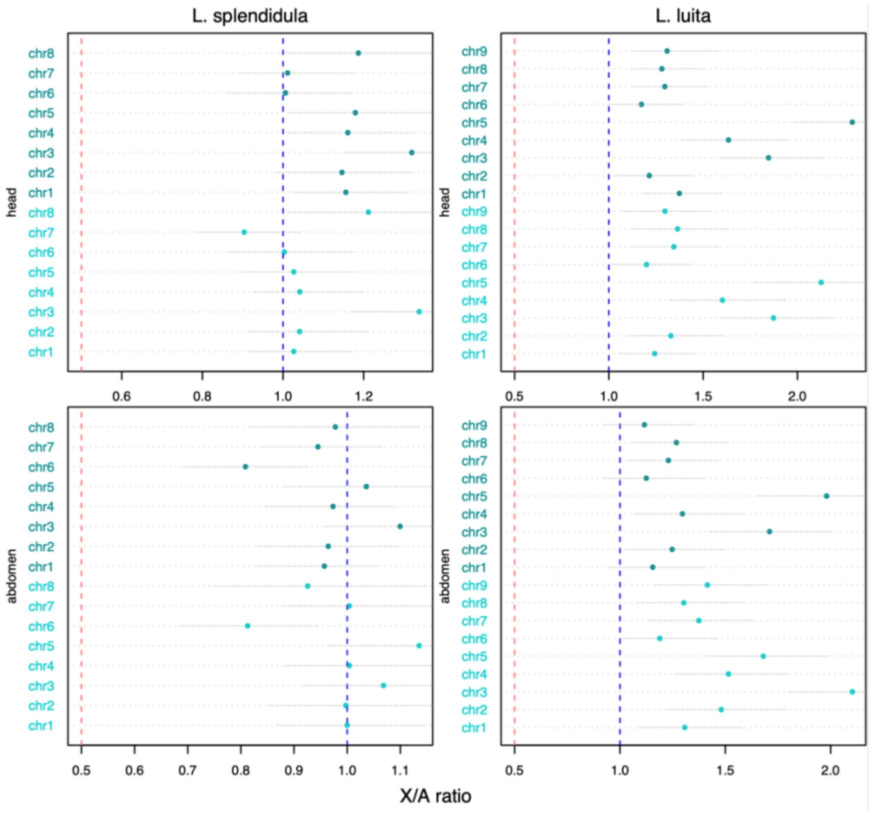

Figure S7. Gene expression ratios between the X and the autosomal (A) chromosomes in head and abdomen tissue of *L. splendidula* and *L. italica*, shown for females (light blue) and males (dark blue).

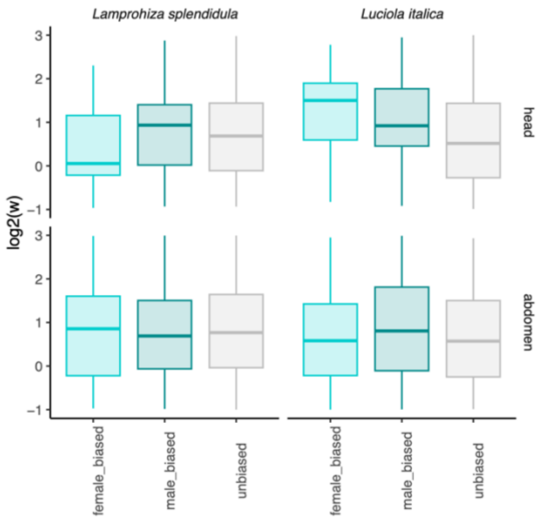

Figure S8. Branch-site model to evaluate differences in protein evolutionary rate between sex- and unbiased genes.
