# Supplementary material for: Two novel genomes of fireflies with different degrees of sexual dimorphism reveal insights into sex-biased gene expression and dosage compensation
Source: Commun Biol. 2024 Jul 27;7:906. doi: 10.1038/s42003-024-06550-6 (PMC11283472; doi:10.1038/s42003-024-06550-6)
Supplement: Supplementary file 3 — Description of Additional Supplementary Files [file 42003_2024_6550_MOESM3_ESM.pdf]

Description of Additional Supplementary Files

File name: Supplementary Data 1
Description: Lamprohiza splendidula head EdgeR analysis for the indetification of sex-biased genes.

File name: Supplementary data 2
Description: Lamprohiza splendidula abdomen EdgeR analysis for the indetification of sex-biased genes.

File name: Supplementary Data 3
Description: Luciola italica head EdgeR analysis for the indetification of sex-biased genes.

File name: Supplementary Data 4
Description: Luciola italica abdomen EdgeR analysis for the indetification of sex-biased genes.

File name: Supplementary Data 5
Description: Data availability submitted to SRA.
